# Editorial: Spinal Muscular Atrophy: Evolutions and Revolutions of Modern Therapy

**DOI:** 10.3389/fneur.2020.00783

**Published:** 2020-07-28

**Authors:** Richard S. Finkel, Ulrike Schara-Schmidt, Tim Hagenacker

**Affiliations:** ^1^Experimental Neurotherapeutics Program, St. Jude Children's Research Hospital, Memphis, TN, United States; ^2^Department of Pediatric Neurology, Developmental Neurology and Social Pediatrics, University Hospital Essen, Essen, Germany; ^3^Department of Neurology, University Hospital Essen, Essen, Germany

**Keywords:** SMA, biomarker, SMN-independent, CSF, radiation exposure, bite force, non-motor features

Classic 5q-linked spinal muscular atrophy (SMA) is an autosomal recessive, progressive motor neuron disorder which affects mainly infants (severe impairment, types I and II) and children (moderate impairment, type III) (1). SMA has been transformed in recent years from a feared and often deadly disease to one where clinicians now have therapeutic options to present patients and parents. Nusinersen and onasemnogene abeparovec increase SMN protein levels in target motor neurons. Results from clinical trials of these drugs demonstrated increased survival and stabilization or improvement in motor and respiratory function (2–4). As such, it is now anticipated that many of these patients living with SMA will survive into adulthood, with some degree of motor function to enable at least limited activity within the home setting and community (5). Adult-onset SMA (type IV) requires more attention by clinicians to better understand how these patients with milder impairment respond to the new drugs (6). With these treatment options, SMA now fulfills the criteria for population-based testing, prompting addition of SMA to the newborn screening panels in several countries or states/regions (7, 8). Pre-symptomatic newborns with genetically determined SMA can now be identified and started on medication that eliminates or delays the onset of symptoms (9). Over time, the clinician will increasingly face two populations of SMA: the pre-symptomatic newborn treated within the first few weeks of life and the symptomatic older infant, child, and adult. Each group of patients will respond differently to these drugs and the expectations will need to be aligned accordingly (10, 11). Counseling parents and parents will be more complex as additional drugs for treatment of SMA are approved by regulatory authorities. Comparative studies will take several years to answer the many questions raised by patients, their caregivers, clinicians, payers for these expensive drugs, and regulatory authorities.

In this issue of Frontiers in Medicine, devoted entirely to SMA, six manuscripts provide a variety of new insights into the rapidly evolving understanding of the disease and its treatment.

A fundamental question of the basic biology of SMA and its pathogenesis is addressed by Hensel et al. in their review focusing upon SMN-independent pathways. While SMN restoration in motor neurons is the fundamental target in SMA, both cell-autonomous and non-cell-autonomous SMN-independent processes appear to be important in mouse models of SMA and also to some degree in symptomatic patients. Understanding how these signaling pathways are relevant in dysfunctional neurons and non-neuronal tissues may provide other treatment opportunities. The authors present two figures that nicely illustrate their concepts and recommendations for the study of potential therapeutic agents: utilize downstream SMA-specific pathways, consider the network of signal pathway nodes especially in disease-relevant tissues and cell types, and study these in combination with SMN-enhancing drugs at appropriate dose levels.

The manuscript by Günther et al. moves this discussion of non-neuronal aspects of SMA into the clinic. They examined patient-reported non-motor symptoms in 70 adult SMA type II and type III patients, and in 59 age-matched control patients. By using a self-reported questionnaire, the authors report a low-level of non-muscle symptoms that was not significantly higher than in the control group. Nor was there an association of the 30 items queried with the severity of motor impairment. This is an important observation as we treat patients with these newer drugs and observe for non-neuromuscular aspects of SMA to emerge, and for identification of off-target side-effects.

The report by Kariyawasam et al. presents a review of the various biomarkers that have been studied in patients with SMA. Genetic factors—including SMN2 copy number, modifier genes such as PLS3 and COROC1, and miRNA—are under active study currently. The authors discuss the potential utility of blood levels of SMN protein and neurofilament levels, electrophysiological data (CMAP, MUNE, decremental response), and muscle imaging (MRI, ultrasound) data as prognostic for the course of disease, predictive of response to medication, and as a pharmacodynamic indicator in response to treatment. These biomarkers will prove invaluable as investigators work to better understand why patients with SMA differ in their rate of progression of disease and response to therapy.

Two manuscripts address nusinersen treatment related issues. The report by Wurster et al. describes CSF findings in patients administered this anti-sense oligonucleotide intrathecally as per current dosing guidelines. Sixty patients, with SMA types I, II, and III, age 7 to 60 years had stored CSF samples analyzed retrospectively. No significant pleocytosis or elevation in total protein, albumen or lactate levels were observed. While reassuring, the authors suggest routine monitoring of CSF remains important as a means of identifying future adverse effects. The report by Kizina et al. addresses the important topic of radiation exposure in patients with SMA who undergo repeated fluoroscopy-guided lumbar punctures for administration of nusinersen. They studied 15 patients with SMA types II and III who underwent this procedure. The effective dose of radiation was higher in patients with complex spine and decreased overall with repeated procedures. No relationship was noted between dosimetry and severity of disease. Routine monitoring of dosimetry with each procedure is recommended.

The report by Kruse et al. describes longitudinal measurements of maximum bite force in two adult SMA female patients with severe SMA type II and minimal limb function. As expected, bite force was significantly reduced as compared to values in healthy adult females. Notably, the values improved during the loading doses then decreased during the 4-month maintenance period until the next dose was administered. These observations suggest that measurement of bite force is a sensitive indicator of response to nusinersen even in very weak adult patients. A prospective longitudinal study is proposed to examine bite force and fatigue. This test measurement could become a standard outcome measure in future clinical trials and also serve as a useful measure of bulbar function in the clinic.

In summary, these six manuscripts addressed diverse but complementary topics in the pathobiology of SMA and the response to treatment. The field is rapidly advancing, with these contributions timely and quite relevant.

## Author Contributions

All authors listed have made a substantial, direct and intellectual contribution to the work, and approved it for publication.

## Conflict of Interest

RF has received institutional support for the conduct of clinical trials in SMA from AveXis, Biogen, Roche and Scholar Rock; has participated in advisory boards from AveXis, Biogen, and Roche; and has received licensing/royalty payments from The Children's Hospital of Philadelphia and Elsevier. US-S recieves speaker and advisory board honoraria from Avexis, Biogen, and Roche. TH receives research support, speaker, and advisory board honoraria from Biogen.
